# Missing value estimation for DNA microarray gene expression data by Support Vector Regression imputation and orthogonal coding scheme

**DOI:** 10.1186/1471-2105-7-32

**Published:** 2006-01-22

**Authors:** Xian Wang, Ao Li, Zhaohui Jiang, Huanqing Feng

**Affiliations:** 1 Department of Electronic Science and Technology, University of Science and Technology of China, USTC, Hefei, P R, China

## Abstract

**Background:**

Gene expression profiling has become a useful biological resource in recent years, and it plays an important role in a broad range of areas in biology. The raw gene expression data, usually in the form of large matrix, may contain missing values. The downstream analysis methods that postulate complete matrix input are thus not applicable. Several methods have been developed to solve this problem, such as K nearest neighbor impute method, Bayesian principal components analysis impute method, *etc*. In this paper, we introduce a novel imputing approach based on the Support Vector Regression (SVR) method. The proposed approach utilizes an orthogonal coding input scheme, which makes use of multi-missing values in one row of a certain gene expression profile and imputes the missing value into a much higher dimensional space, to obtain better performance.

**Results:**

A comparative study of our method with the previously developed methods has been presented for the estimation of the missing values on six gene expression data sets. Among the three different input-vector coding schemes we tried, the orthogonal input coding scheme obtains the best estimation results with the minimum Normalized Root Mean Squared Error (*NRMSE*). The results also demonstrate that the SVR method has powerful estimation ability on different kinds of data sets with relatively small *NRMSE*.

**Conclusion:**

The SVR impute method shows better performance than, or at least comparable with, the previously developed methods in present research. The outstanding estimation ability of this impute method is partly due to the use of the most missing value information by incorporating orthogonal input coding scheme. In addition, the solid theoretical foundation of SVR method also helps in estimation of performance together with orthogonal input coding scheme. The promising estimation ability demonstrated in the results section suggests that the proposed approach provides a proper solution to the missing value estimation problem. The source code of the SVR method is available from  for non-commercial use.

## Background

Gene expression profiles, produced by the microarray experiments, provide a way to investigate the expression levels of thousands of genes under various experimental conditions. It has been used in a broad range of areas in biology, such as regulatory pathway inferring, functional gene finding, *etc*. [1-6]. The downstream processing methods, such as clustering [7,8], supervised learning algorithms [9-11], *etc*., have been applied to the analysis of the available data.

Consisting of hundreds or even thousands of gene-specific DNA sequences, gene expression microarrays produce massive gene expression data sets in the form of large matrices, which, however, contain the missing values. These missing values can be caused by various factors, such as insufficient resolution, image corruption, or simply due to dust or scratches on the slide. Moreover, systematically data missing might also present in the robotic method for the generation of gene expression profiles.

Repetition of identical experiments [12] has been adopted to validate downstream microarray analysis algorithms dealing with the missing value issue. However, this method is costly and time consuming. The naïve ways that have been commonly used include omitting the expression profile vector with missing values, and padding them with zeros, or row averages [13]. These methods are widely used by biologists, but the disadvantages of them are obvious: omitting the profile vector results in losing useful information; padding them with zeros and row averages do not provide proper missing value estimation. Rather sophisticated approaches have been proposed by Troyanskaya *et al*. [12]. The approaches are based on K-nearest neighbor algorithm (KNN impute) and Singular Value Decomposition algorithm (SVD impute). The KNN impute method aims at finding *k *genes mostly similar to genes containing missing values, where the similarity is estimated by Euclidean distance measure, and the missing values are imputed with values of weighted average from these neighboring genes. The SVD impute method obtains a set of mutually orthogonal expression patterns (eigengenes) from the gene expression matrix, and impute the missing values by regressing the gene against the k eigengenes and linearly combining the eigengenes. In most cases, the KNN imputing performs better and more robust than does the SVD, which is also good on time series data corrupted by low level noise. These initial attempts of imputing the missing-values by means of mathematical fashion have shown the promising progress in terms of superior performance of estimation accuracy on the test datasets.

Recently, Oba *et al*. has developed an optimization method based on Bayesian principal component analysis (BPCA impute) [14], which outperforms the KNN and the SVD impute methods. One of the features, which allow the method provides better performances, is the capability of auto-selection of the parameters used in estimation. The method also claims better estimation performance when the number of the samples is large. Bayesian variable selection algorithm, developed by Zhou *et al*. [15], also aims at the auto-selection of the number of the nearest neighboring genes used in estimation. In this algorithm, both linear and nonlinear regressions are used for the estimation rule, and the procedures for the fast implementation have been developed for the essential steps of the algorithm. Kim *et al*. [16] has proposed a method based on local least squares (LLS impute), which exploited the local similarity structures in the data as well as the least squares optimization process. Some of these methods, however, did not take most use of missing values in one row of certain expression profile (see method section), so that other missing values are just excluded, or padded with zeros or row average in the estimation.

In this paper, we propose a new approach based on the Support Vector Regression (SVR) to estimate the missing values and use orthogonal input coding scheme to address the issue of multiple missing values in one row of certain expression profile. To evaluate the proposed method, six microarray datasets have been tested with various parameter settings. The superior performance, comparing with KNN, BPCA, and LLS impute methods, indicates the promising estimation ability of the method.

In this paper, we use *D *∈ *R*^m*n ^to denote the whole gene expression data matrix, where *m *is the number of genes, and *n *is the number of different experimental conditions, i.e., entry *d*_*i*, *j *_in the expression matrix denotes the expression level of the i-th gene in the j-th experimental condition.

## Results

### Data sets

In this paper, the performance of each method is evaluated by using six data sets. The first two data sets, initially made available by Spellman *et al *[17], focus on identification of the cell-cycle regulated genes in yeast *Saccharomyces cerevisiae*, and are all time series data sets. One of the data set is from the study of α-factor block experiments, and it contains 18 sampling points of each gene. This data set is referred as data A hereafter. And another data set, referred to as data E, is an elutriation dataset with 14 sampling points. The third data set, data G, is from Gasch's experiments [18] focusing on the response to the environment changes of genes in yeast, and is a data set containing 173 expression ratios for each gene. After removing all the columns with more than 8% missing entries, we select all gene rows without the missing values. Among the resulting 2990 gene rows, we randomly select 44 columns to construct a non-time series subset of the whole data set by rearranging the columns at random. The fourth data set, data I, is original cDNA microarray data relevant to human colorectal cancer (CRC) [19]. This data set contains 205 primary CRCs that include 127 non-metastatic primary CRCs, 54 metastatic primary CRCs to the liver, and 12 normal colonic epithelia that have been histopathologically confirmed to be free of cancer [14]. In this paper, the total number of the genes used from this subset is 758. The fifth data set, data P, is a gene expression data set relevant to the molecular pharmacology of cancer, which contains gene expression profiles in 60 human cancer cell lines in a drug discovery screen [20]. This data set contains 9706 genes with 60 sampling points for each gene. After removing all the columns with more than 30% missing entries, and selecting all gene rows without missing entries, we finally get the data set with 4508 genes, which contain 31 sampling points for each gene. The last data set, data C, is also from reference [17], the same data set used in Kim's paper, focusing on the cell-cycle-regulated genes. This data set was classified into five classes by Spellman, from the same 14 experiments as in data E. Data C is used to test how much an imputing method is able to take advantage of strongly correlated genes in estimating the missing values.

The data sets we used in our study are processed with several steps. Firstly, they are log-transformed after they are taken from the image. Secondly, the rows and the columns which contain much missing values are excluded. Thirdly, before using orthogonal input coding scheme and SVR impute method, each of the columns are scaled to between 0 and 1, which means the data sets are normalized. Mean-normalizing the data will further help in regression performance using Euclidian Distance. Finally, the data sets with all these pre-procession are used to construct the "complete matrix".

### Measurements for performance

We constructed the "complete matrices" by removing all the rows containing raw missing values, and randomly created the artificial missing values, from 1% to 20%, of the entries in a matrix. The performance is measured by the Normalized Root Mean Squared Error (*NRMSE*), defined as

NRMSE=mean[(yguess−yanswer)2]variance[yanswer]     (1)
 MathType@MTEF@5@5@+=feaafiart1ev1aaatCvAUfKttLearuWrP9MDH5MBPbIqV92AaeXatLxBI9gBaebbnrfifHhDYfgasaacH8akY=wiFfYdH8Gipec8Eeeu0xXdbba9frFj0=OqFfea0dXdd9vqai=hGuQ8kuc9pgc9s8qqaq=dirpe0xb9q8qiLsFr0=vr0=vr0dc8meaabaqaciaacaGaaeqabaqabeGadaaakeaacqWGobGtcqWGsbGucqWGnbqtcqWGtbWucqWGfbqrcqGH9aqpdaGcaaqaamaalaaabaGaemyBa0MaemyzauMaemyyaeMaemOBa4Maei4waSLaeiikaGIaemyEaK3aaSbaaSqaaiabdEgaNjabdwha1jabdwgaLjabdohaZjabdohaZbqabaGccqGHsislcqWG5bqEdaWgaaWcbaGaemyyaeMaemOBa4Maem4CamNaem4DaCNaemyzauMaemOCaihabeaakiabcMcaPmaaCaaaleqabaGaeGOmaidaaOGaeiyxa0fabaacbiGae8NDayNae8xyaeMae8NCaiNaemyAaKMaemyyaeMaemOBa4Maem4yamMaemyzauMaei4waSLaemyEaK3aaSbaaSqaaiabdggaHjabd6gaUjabdohaZjabdEha3jabdwgaLjabdkhaYbqabaGccqGGDbqxaaaaleqaaOGaaCzcaiaaxMaadaqadaqaaiabigdaXaGaayjkaiaawMcaaaaa@6D1C@

Where the mean and the variance are calculated from the complete matrix, and *y*_*guess *_are the estimated vectors for the missing values in the matrix while *y*_*anwer *_are the true value vectors for the artificial missing values. The *NRMSE *varies according to the estimation performance. When predicted values are accurate, the *NRMSE *reaches its minimum value 0, and when the prediction is very poor or the noise involved is too large, the *NRMSE *becomes much larger.

### Input coding scheme selection

**Figure 1:**
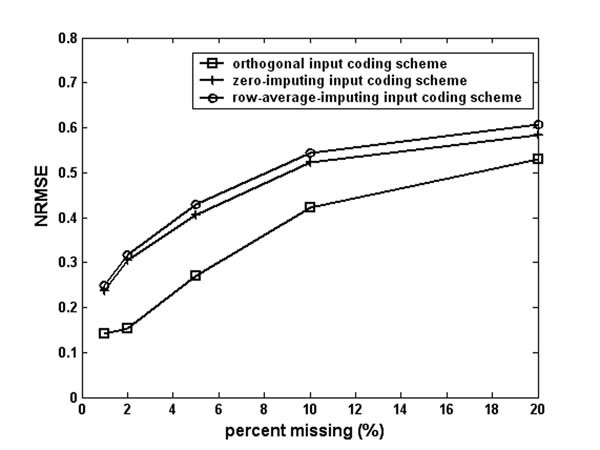
**Influence of different input coding schemes on regression performance**. The percentage of entries missing in the 'complete matrix' and the *NRMSE *of each input coding scheme are shown in the horizontal and vertical axes, respectively.

Figure 1 shows the comparison of performance. The horizontal and vertical axes indicate the percentage of entries missing in the 'complete matrix' and the *NRMSE *of each input coding scheme, respectively. In the Figure 1, the results reveal that orthogonal input coding scheme outperforms the other two input coding schemes, and the performances of the zero-imputing and the row-average-imputing input coding scheme are quite similar. For example, when the percentage of entries missing is 20%, the *NRMSE *of the orthogonal input coding scheme reaches 0.5269, and the *NRMSE *of the other two input coding schemes are 0.5841 and 0.6064, respectively. When the percentage of entries missing is 1%, the *NRMSE *of the orthogonal input coding scheme goes as low as 0.1176, which is much lower than the *NRMSE *of the other two input coding scheme, 0.2384 and 0.2483.

### Performance comparison with other methods

**Figure 2:**
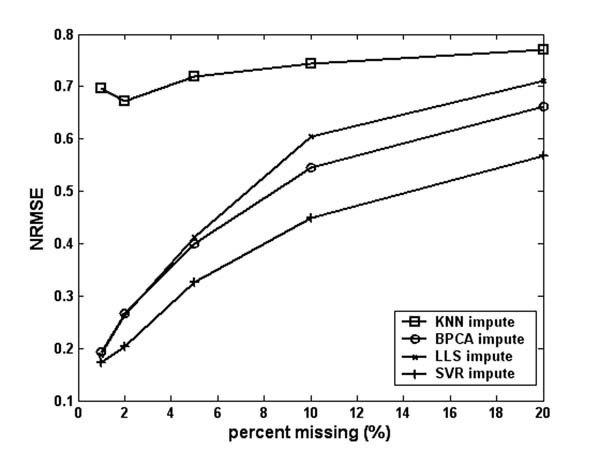
**Performance of the four methods on data A**. The percentage of entries missing in the 'complete matrix' and the *NRMSE *of each missing value estimation method are shown in the horizontal and vertical axes, respectively.

**Figure 3:**
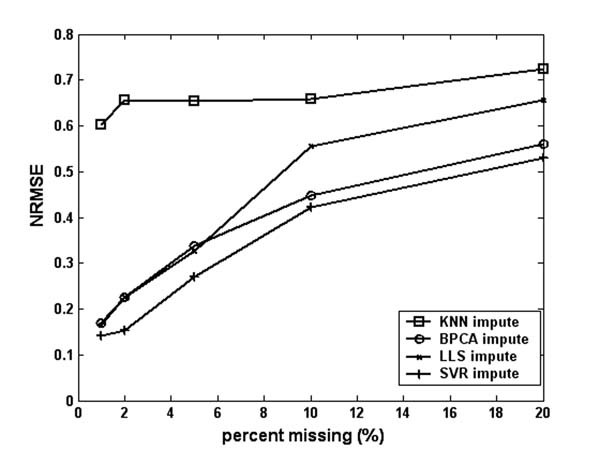
Performance of the four methods on data E.

**Figure 4:**
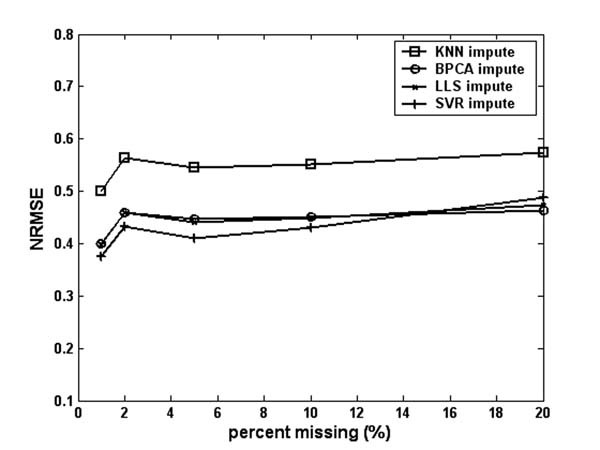
Performance of the four methods on data G.

**Figure 5:**
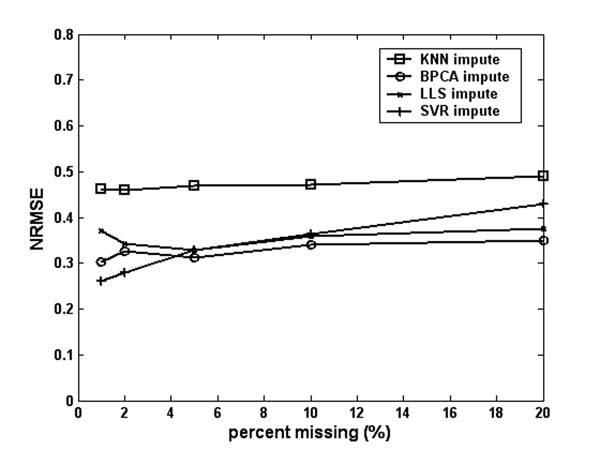
Performance of the four methods on data I.

**Figure 6:**
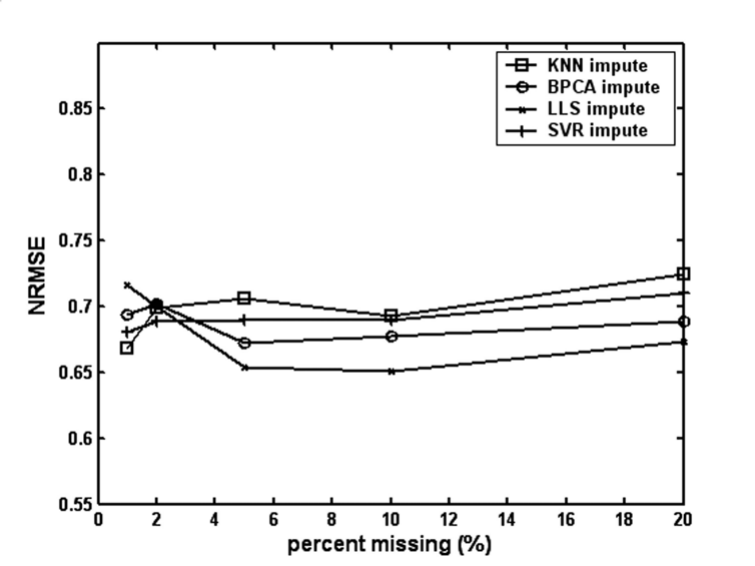
Performance of the four methods on data P.

**Figure 7:**
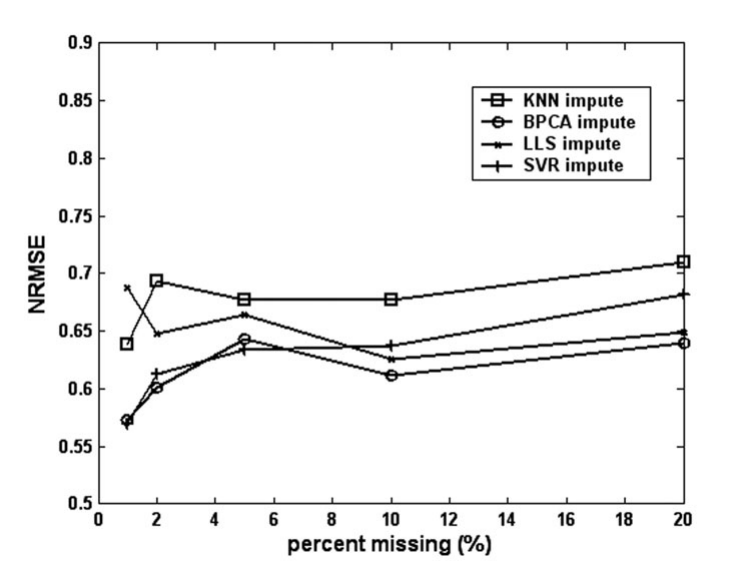
Performance of the four methods on data C.

The performance of the SVR impute method, assessed over five different data sets, has been compared with three imputing approaches, i.e., KNN, BPCA and LLS impute method. The k-value in the KNN impute method was preset as 15, according to proposed scope of between 10 and 20. The parameter sets for the BPCA impute method were taken directly from published resource. The LLS impute method is a non-parameter method, and the referenced programs were used. Performance of each method on different data sets is shown in Figure 2, 3, 4, 5, 6, 7.

Data A and data E, which are the noisy time-series data sets, were pre-processed by removing all genes containing the missing values. They produce complete matrix with 4304 genes. From Figure 2 and 3 we can see that the SVR impute method notably outperforms the other three methods on these two data sets. And we obtain similar results on data A. The SVR also performs stably across the data missing percentages.

As claimed by Troyanskaya [12], data G is the most challenging prediction data set, where a clear expression pattern is often absent. The complete matrix contains 2990 genes after pre-pressing. Figure 4 shows that among all four methods, in most cases, the SVR method gets minimal NRMSE. When percentage of missing values in the data sets is below 20%, the SVR achieves the best result. And when percentage of missing values reaches 20%, the NRMSE of the SVR is a little larger than those of the BPCA and the LLS impute methods, and still much smaller than that of the KNN impute method. This shows the SVR method is comparable with, if not better than, the previous methods on non time series data set.

Data I is a data set relevant to human cancer, which involves much more complex regulation mechanisms. Therefore, this type of gene expression profile data set is much difficult for the missing value estimation. Figure 5 shows the performance of four methods on data I. On this data set, the SVR method gets similar results as it does on data G. When the percentage of missing values is below 10%, the SVR method gets good result. While the percentage of missing values exceeds 10%, the NRMSE of SVR is a little larger. On this data set, our method shows comparable estimating ability with the previous methods.

Relevant to many kinds of human cancers, including colorectal, renal, ovarian, breast, prostate, lung and central nervous system origin, as well as leukaemias and melanomas, data P becomes the most difficult data set for missing value estimation. Figure 6 shows the performance of each method on this data set. On this data set, the SVR impute method gets similar result as other previous methods. All the methods get similar estimate performance with the NRMSE between 0.65 and 0.7. On this data set, the SVR impute method performs robustly as the percentage of the missing values increase.

Data C is designed to test how much an imputing method is able to take advantage of strongly correlated genes in estimating the missing values according to the research work by Kim *et al*. [16] We can see from Figure 7 that the SVR method gets similar result as other previous methods. This indicates the SVR method can take better use of strongly correlated genes than do other three methods in estimating the missing values.

### Performance of SVR method on dataset with higher noise levels

**Figure 8:**
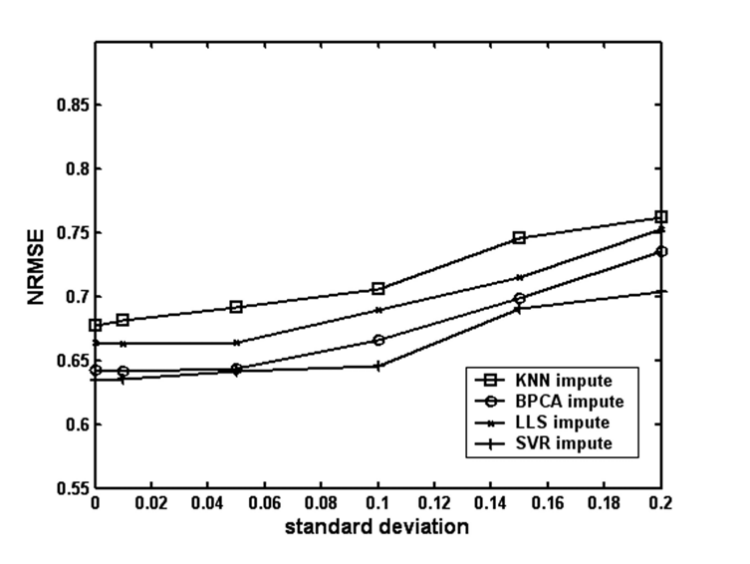
**Comparison of the NRMSEs with respect to noise levels**. Artificial noise, with normal distribution of a mean μ = 0 and various standard deviations (σ = 0.01,0.05,0.1,0.15,0.2), were added to data C to construct the five noisy datasets.

For the real data set that always contains noise caused by various reasons, a good estimation method must be robust against certain levels of noise. To test the robustness of the SVR method, we prepare five noisy datasets by adding random noise of various levels, with normal distribution, on data C, as has been proposed by Kim [16]. To generate the six datasets, we first build six noise matrices with normal distribution of zero-mean (μ = 0) and various standard deviations (σ = {0.01, 0.05, 0.10, 0.15 0.20}), and then add them to data C with 5% entries missing. From Figure 8 we can see that the performance of the SVR method is not very sensitive to the noise level, especially when σ is less than 0.15. Therefore, the SVR method is more robust against noise.

## Discussion

### Performance compared with previous methods

Three pervious methods are used to compare the performance of the SVR impute method in our research. One of the advantages of the SVR method is that it makes most use of the information from the original data sets. The orthogonal input coding scheme raises the estimation performance notably, which contributes to the best performance of the SVR method among these four methods. In the case of the KNN and the LLS method, the redundant missing values in the samples or genes with many missing values are just neglected, while the BPCA method simply regards them equally with the non-missing values. Another advantage comes from the SVR method itself. The SVR method is a method based on the structural risk minimization principle in statistical learning theory, which guarantees the global optimal solution of the dual quadratic programming problem. The KNN method linearly combines the similar genes by weighting the average values of them. The coefficients used in combination are calculated by using Euclidean distance, which is not an optimal measurement for gene similarity. This makes the KNN method perform worst among all the methods. The BPCA method uses the principal component regression, which makes the results highly depend on the numbers of the principal axes. If genes have dominant local similarity structures, the result of this method may not be the global optimal. The LLS method is a method based on local similar structure. It shares the similar linear combination of k nearest genes as the KNN impute, and surpasses the KNN impute by optimizing the coefficients of the non-missing part of the similar genes using the least square solution. The LLS method is based on local similarity structure of the data set, which draws back its performance when the local similarity is not very clear. In most cases, LLS method performs worse than BPCA impute method and SVR impute method.

Besides yeast gene expression profiles, our method also works well on the data sets those are much more difficult for regression, because of the complex regulation mechanisms involved (Figure 5 and Figure 6). What's more, the length of the expression profiles in data I is 205, which is much larger than the data sets relevant to yeast. This might make it more complex for regression. Figure 5 and Figure 6 show that the SVR method achieves comparative results to the previous methods. When the percentage of missing values becomes too large, the SVR impute method performs little worse than do the BPCA and the LLS impute method. This is partly due to the grid search strategy for the parameter sets. To maintain proper parameter sets, the user should specify the range of the parameters been searched, so the parameter sets might not be the optimum. The parameter selection is also a problem that has to be solved in the Support Vector Regression. Even if the parameter set might not be the optimum, the result is still comparative with other impute methods. Thus the SVR impute method performs well in present research.

### Input coding scheme selection

The main difference between the orthogonal input coding scheme and the other two is that the former utilizes the most information in the whole matrix, while the latter does not. All the values in two non-orthogonal input schemes are regarded equally in the input vector, which is not true. The flag bits in orthogonal coding, on the other hand, mark its strength by taking the missing value information into account, which is able to represent the difference between the missing and non-missing values.

Let *x*_1 _= (*x*_11_, *x*_12_, *x*_13_) denotes the gene expression profile, where *x*_12 _and *x*_13 _are missing. When imputing *x*_13_, the orthogonal input coding schemes gets the input vector of *vector*_*orthogonal *_= (*x*_11_, 0, 0, 1) (see method), and the zero-imputing input coding scheme gets the input vector of *vector*_*zero *_= (*x*_11_, 0). When calculation the kernel function *K*(*x*_*i*_, *x*_*j*_) = exp(-*γ*||*x*_*i *_- *x*_*j*_||^2^) used in final regression function (9), we get:

*k*_*orthogonal*_(*x*_1_, *x*_*i*_) = exp(-*γ*(*x*_11_^2 ^+ 1))*exp(-*γ*(*x*_12_^2 ^+ *x*_22_^2^))*exp(2*x_11_*x_12_)     (2)

in the orthogonal input coding scheme and

*k*_*zero*_(*x*_1_, *x*_*i*_') = exp(-*γ*(*x*_11_^2^))*exp(-*γ*(*x*_12_^2 ^+ *x*_22_')^2^))*exp(2*x_11_*x_12_)     (3)

in the zero-imputing input coding scheme, where *x*_*i *_= (*x*_21_, *x*_22_, *x*_23_), *x*_*i*_' = (*x*_21_', *x*_22_', *x*_23_') denote the center point of the SVR in the orthogonal input coding scheme and the zero-imputing input coding scheme during calculation, respectively. The difference of the two input coding scheme in the kernel function is not only the difference of the center point, but also a coefficient of exp(-*γ*). So they perform differently in regression performance. It is also indicated that why the value of flag bit is set to be 1, but not other values. The flag bit in the orthogonal input coding scheme is used as the coefficient of the parameter *γ *in the kernel function and the final regression function. Since the parameter *γ *can be tuned by the user, the flag bit can be safely set to be 1 without any influence on the final regression result.

## Conclusion

In this paper, we introduce the Support Vector Regression (SVR) impute as a novel method for estimation of the missing values in gene expression profile. Testing results reveal that the SVR impute has outstanding prediction ability in the estimation of the missing values problem and robust against the noise. Moreover, our approach makes most use of the missing value information in the whole gene expression matrix by using orthogonal input coding scheme. A comprehensive comparison of *NRMSE *on five data sets shows that the SVR impute performs comparative with, if not better than, the other missing value estimation methods in this area ever since, and it appears to be a proper solution to the missing value estimation in gene expression profile.

## Methods

### Support Vector Regression

Support Vector machine (SVM), which is based on the structural risk minimization principle in statistical learning theory, is a powerful tool for general purpose machine learning problem [21]. It solves the "over-fitting" problem by using structure risk minimization principle, which minimizes both empirical risk and confidence interval. In practice, two kinds of SVMs are provided for different purpose: Support Vector machine for classification (SVC) and Support Vector machine for regression (SVR). The SVC has been extensively investigated in the areas of bioinformatics, such as enzyme family classification [22], protein secondary structure prediction [23], and protein relative solvent accessibility prediction [24], etc., for that it is not only well founded in theory, but also very efficient in practical purpose. As another aspect of the SVM, although the SVR has seldom been used in these areas, the SVC also has shown its powerful ability of resolving problems in our work.

**Figure 9:**
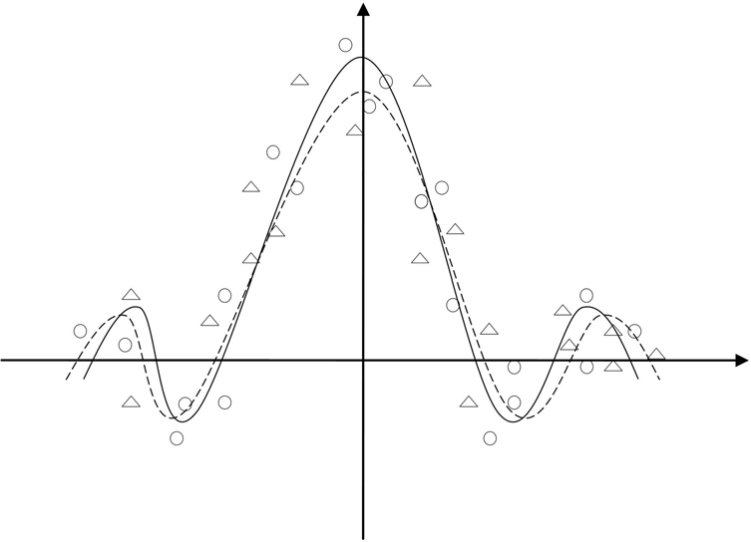
**Illustration of the support vector machine method used for regression**. Given a set of observed training data (circles and triangles), which are sampled from the hidden original function *f*(*x*) (solid line) and may be polluted by noise during this procedure, the SVR constructs the fitted regression function φ(*x*) (dashed line) by solving the corresponding optimal problem with constrains. The support vectors and non-support vectors are denoted with circles and triangles, respectively.

Generally, the SVR is carried out with two steps: first, the SVR maps the samples from the input space with a low dimension into a much higher (sometimes infinite) dimensional space with a kernel function, and then searches for the global optimal solution to the corresponding problem using the quadratic programming. The so called support vectors (Figure 9) are these samples with non-zero Lagrange multiplier. Given a set of observed training data (circles and triangles), which are sampled from the hidden original function *f*(*x*) (solid line) and maybe polluted by noise during this procedure, SVR constructs the fitted regression function φ(*x*)(dashed line) by solving the corresponding optimal problem with constrains. The support vectors and non-support vectors are denoted with circles and triangles, respectively. The support vectors are these input samples that will be further used in regression, which means if we remove all the non support vectors in the data set, the regression result will not be influenced. The mathematical concept of support vectors and how to calculate these support vectors will be discussed later in this section.

The final regression function of the SVR is determined by the support vectors. The number of the support vectors is usually small when compared to the total number of the samples. Let {(*x*_1_, *z*_1_),...,(*x*_*n*_, *z*_*n*_)} denotes a set of the training data, which was sampled from the original function *f*(*x*) and may be polluted by noise during this procedure. Here, *x*_*i *_∈ *R*^*n *^is the input and *z*_*i *_∈ *R*^*l *^is a target output. The standard form of the SVR is

min⁡w,b,ξ,ξ*12WTW+C∑i=1nξi+C∑i=1nξi*     (4)
 MathType@MTEF@5@5@+=feaafiart1ev1aaatCvAUfKttLearuWrP9MDH5MBPbIqV92AaeXatLxBI9gBaebbnrfifHhDYfgasaacH8akY=wiFfYdH8Gipec8Eeeu0xXdbba9frFj0=OqFfea0dXdd9vqai=hGuQ8kuc9pgc9s8qqaq=dirpe0xb9q8qiLsFr0=vr0=vr0dc8meaabaqaciaacaGaaeqabaqabeGadaaakeaadaWfqaqaaiGbc2gaTjabcMgaPjabc6gaUbWcbaGaem4DaCNaeiilaWIaemOyaiMaeiilaWIaeqOVdGNaeiilaWIaeqOVdGNaeiOkaOcabeaakmaalaaabaGaeGymaedabaGaeGOmaidaaiabdEfaxnaaCaaaleqabaGaemivaqfaaOGaem4vaCLaey4kaSIaem4qam0aaabCaeaacqaH+oaEdaWgaaWcbaGaemyAaKgabeaakiabgUcaRiabdoeadnaaqahabaGaeqOVdG3aa0baaSqaaiabdMgaPbqaaiabcQcaQaaaaeaacqWGPbqAcqGH9aqpcqaIXaqmaeaacqWGUbGBa0GaeyyeIuoaaSqaaiabdMgaPjabg2da9iabigdaXaqaaiabd6gaUbqdcqGHris5aOGaaCzcaiaaxMaadaqadaqaaiabisda0aGaayjkaiaawMcaaaaa@5DAE@

Subject to

*W*^*T*^φ(*x*_*i*_) + *b *- *z*_*i *_≤ ε + ξ_*i*_,

*z*_*i *_- *W*^*T*^φ(*x*_*i*_) - *b *≤ ε + ξi*
 MathType@MTEF@5@5@+=feaafiart1ev1aaatCvAUfKttLearuWrP9MDH5MBPbIqV92AaeXatLxBI9gBaebbnrfifHhDYfgasaacH8akY=wiFfYdH8Gipec8Eeeu0xXdbba9frFj0=OqFfea0dXdd9vqai=hGuQ8kuc9pgc9s8qqaq=dirpe0xb9q8qiLsFr0=vr0=vr0dc8meaabaqaciaacaGaaeqabaqabeGadaaakeaaliabe67a4naaDaaameaacqWGPbqAaeaacqGGQaGkaaaaaa@30DF@,

ξ_*i*_, ξi*
 MathType@MTEF@5@5@+=feaafiart1ev1aaatCvAUfKttLearuWrP9MDH5MBPbIqV92AaeXatLxBI9gBaebbnrfifHhDYfgasaacH8akY=wiFfYdH8Gipec8Eeeu0xXdbba9frFj0=OqFfea0dXdd9vqai=hGuQ8kuc9pgc9s8qqaq=dirpe0xb9q8qiLsFr0=vr0=vr0dc8meaabaqaciaacaGaaeqabaqabeGadaaakeaaliabe67a4naaDaaameaacqWGPbqAaeaacqGGQaGkaaaaaa@30DF@ ≥ 0, *i *= 1,...,*n *    (5)

Where *W *is the solution of the primal formulation and *C *is the regulation parameter that controls the trade off between margin and prediction error denoted by ξ_*i*_, ξi*
 MathType@MTEF@5@5@+=feaafiart1ev1aaatCvAUfKttLearuWrP9MDH5MBPbIqV92AaeXatLxBI9gBaebbnrfifHhDYfgasaacH8akY=wiFfYdH8Gipec8Eeeu0xXdbba9frFj0=OqFfea0dXdd9vqai=hGuQ8kuc9pgc9s8qqaq=dirpe0xb9q8qiLsFr0=vr0=vr0dc8meaabaqaciaacaGaaeqabaqabeGadaaakeaaliabe67a4naaDaaameaacqWGPbqAaeaacqGGQaGkaaaaaa@30DF@. φ(*x*_*i*_) is a non-linear function mapping the input feature into a higher dimensional space and ε is the error probability that controls the most deviation of the regression function from the actually obtained target.

The formulation above corresponds to dealing with a so called ε-insensitive loss function:

|ξ|ε:={0,if|ξ|≤ε|ξ|−ε,otherwose     (6)
 MathType@MTEF@5@5@+=feaafiart1ev1aaatCvAUfKttLearuWrP9MDH5MBPbIqV92AaeXatLxBI9gBaebbnrfifHhDYfgasaacH8akY=wiFfYdH8Gipec8Eeeu0xXdbba9frFj0=OqFfea0dXdd9vqai=hGuQ8kuc9pgc9s8qqaq=dirpe0xb9q8qiLsFr0=vr0=vr0dc8meaabaqaciaacaGaaeqabaqabeGadaaakeaadaabdaqaaiabe67a4bGaay5bSlaawIa7amaaBaaaleaacqaH1oqzaeqaaOGaeiOoaOJaeyypa0ZaaiqaaeaafaqaaeGabaaabaGaeGimaaJaeiilaWIaeeyAaKMaeeOzay2aaqWaaeaacqaH+oaEaiaawEa7caGLiWoacqGHKjYOcqaH1oqzaeaadaabdaqaaiabe67a4bGaay5bSlaawIa7aiabgkHiTiabew7aLjabcYcaSiabb+gaVjabbsha0jabbIgaOjabbwgaLjabbkhaYjabbEha3jabb+gaVjabbohaZjabbwgaLbaaaiaawUhaaiaaxMaacaWLjaWaaeWaaeaacqaI2aGnaiaawIcacaGLPaaaaaa@5BF0@

To a certain extent, regulation parameter C controls the complexity of the learning machine, and the training speed is also influenced by this parameter. The number of the support vectors will be influenced by these parameters. Generally, the larger ε is, the less support vectors there need. The corresponding dual quadratic programming problem is

min⁡α,α*12(α−α*)TQ(α−α*)+ε∑i=1l(αi+αi*)+∑i=1lZi(αi−αi*)     (7)
 MathType@MTEF@5@5@+=feaafiart1ev1aaatCvAUfKttLearuWrP9MDH5MBPbIqV92AaeXatLxBI9gBaebbnrfifHhDYfgasaacH8akY=wiFfYdH8Gipec8Eeeu0xXdbba9frFj0=OqFfea0dXdd9vqai=hGuQ8kuc9pgc9s8qqaq=dirpe0xb9q8qiLsFr0=vr0=vr0dc8meaabaqaciaacaGaaeqabaqabeGadaaakeaadaWfqaqaaiGbc2gaTjabcMgaPjabc6gaUbWcbaGaeqySdeMaeiilaWIaeqySdeMaeiOkaOcabeaakmaalaaabaGaeGymaedabaGaeGOmaidaaiabcIcaOiabeg7aHjabgkHiTiabeg7aHjabcQcaQiabcMcaPmaaCaaaleqabaGaemivaqfaaOGaemyuaeLaeiikaGIaeqySdeMaeyOeI0IaeqySdeMaeiOkaOIaeiykaKIaey4kaSIaeqyTdu2aaabCaeaacqGGOaakaSqaaiabdMgaPjabg2da9iabigdaXaqaaiabdYgaSbqdcqGHris5aOGaeqySde2aaSbaaSqaaiabdMgaPbqabaGccqGHRaWkcqaHXoqydaqhaaWcbaGaemyAaKgabaGaeiOkaOcaaOGaeiykaKIaey4kaSYaaabCaeaacqWGAbGwdaWgaaWcbaGaemyAaKgabeaakiabcIcaOiabeg7aHnaaBaaaleaacqWGPbqAaeqaaOGaeyOeI0IaeqySde2aa0baaSqaaiabdMgaPbqaaiabcQcaQaaakiabcMcaPiaaxMaacaWLjaWaaeWaaeaacqaI3aWnaiaawIcacaGLPaaaaSqaaiabdMgaPjabg2da9iabigdaXaqaaiabdYgaSbqdcqGHris5aaaa@73A3@

Subject to

∑i=1l(αi−αi*)=0,0≤αi,αi*≤C,i=1,…l     (8)
 MathType@MTEF@5@5@+=feaafiart1ev1aaatCvAUfKttLearuWrP9MDH5MBPbIqV92AaeXatLxBI9gBaebbnrfifHhDYfgasaacH8akY=wiFfYdH8Gipec8Eeeu0xXdbba9frFj0=OqFfea0dXdd9vqai=hGuQ8kuc9pgc9s8qqaq=dirpe0xb9q8qiLsFr0=vr0=vr0dc8meaabaqaciaacaGaaeqabaqabeGadaaakeaadaaeWbqaaiabcIcaOiabeg7aHnaaBaaaleaacqWGPbqAaeqaaOGaeyOeI0IaeqySde2aa0baaSqaaiabdMgaPbqaaiabcQcaQaaakiabcMcaPiabg2da9iabicdaWiabcYcaSiabicdaWiabgsMiJkabeg7aHnaaBaaaleaacqWGPbqAaeqaaOGaeiilaWIaeqySde2aa0baaSqaaiabdMgaPbqaaiabcQcaQaaakiabgsMiJkabdoeadjabcYcaSiabdMgaPjabg2da9iabigdaXiabcYcaSiablAciljabdYgaSjaaxMaacaWLjaWaaeWaaeaacqaI4aaoaiaawIcacaGLPaaaaSqaaiabdMgaPjabg2da9iabigdaXaqaaiabdYgaSbqdcqGHris5aaaa@5933@

Where *Q*_*ij *_= *K*(*x*_*i*_, *x*_*j*_) = φ(*x*_*i*_)^*T *^φ(*x*_*j*_). The final regression function can be expressed as

φ(x)=∑i=1l(−αi+αi*)K(xi,x)+b     (9)
 MathType@MTEF@5@5@+=feaafiart1ev1aaatCvAUfKttLearuWrP9MDH5MBPbIqV92AaeXatLxBI9gBaebbnrfifHhDYfgasaacH8akY=wiFfYdH8Gipec8Eeeu0xXdbba9frFj0=OqFfea0dXdd9vqai=hGuQ8kuc9pgc9s8qqaq=dirpe0xb9q8qiLsFr0=vr0=vr0dc8meaabaqaciaacaGaaeqabaqabeGadaaakeaacqaHgpGzcqGGOaakcqWG4baEcqGGPaqkcqGH9aqpdaaeWbqaaiabcIcaOiabgkHiTiabeg7aHnaaBaaaleaacqWGPbqAaeqaaaqaaiabdMgaPjabg2da9iabigdaXaqaaiabdYgaSbqdcqGHris5aOGaey4kaSIaeqySde2aa0baaSqaaiabdMgaPbqaaiabcQcaQaaakiabcMcaPiabdUealjabcIcaOiabdIha4naaBaaaleaacqWGPbqAaeqaaOGaeiilaWIaemiEaGNaeiykaKIaey4kaSIaemOyaiMaaCzcaiaaxMaadaqadaqaaiabiMda5aGaayjkaiaawMcaaaaa@526D@

And *K*(*x*_*i*_, *x*) is the kernel function, which can be set in different forms, such as polynomial kernel function, radial basis kernel function, sigmoid kernel function, etc. The support vectors are those input vectors with the corresponding α of non-zero value.

### Parameter sets for SVR

Performance of the SVR depends on its kernel functions and corresponding parameter sets. Among different kinds of the kernel functions, we choose radial basis function for its outstanding performance and relatively short operation time. The formulation of the radial basis function is as follows:

*K*(*x*_*i*_, *x*_*j*_) = exp(-*γ*||*x*_*i *_- *x*_*j*_||^2^)     (10)

*γ *is a parameter that can be designed by user. The toolkit for the SVR implementation we choose is *LibSVM *[25].

Three parameters, *C*, *γ *and *ε*, can be tuned for this kernel function as has been defined before. To get optimum parameter sets for the SVR, a grid search strategy is performed over the training data set. All the profiles without missing data in the certain column are used to construct the training data set. We then apply a grid search strategy with 5 fold cross-validation over the training data set for each column individually, where the ranges of the parameters are specified by the user. Finally, the parameter set with best performance on the training data set over all the columns in the data set is chosen for the SVR method.

The time the program takes depends on the size of the data set and the parameter sets, the larger the C parameter is, the slower the program runs. Usually, it will finish regression in several minutes. For example, on data set C, the regression progress takes 2.85 seconds, while on data set P, the regression progress takes 595.78 seconds when using the same parameter set. When searching for the parameter sets using grid search scheme, the time cost depends on several factors: firstly, the size of the data set. The larger the data set is, the longer it will take. Secondly, the range of the parameter sets, which was assigned by the user. As we use 5 fold cross-validation in search scheme, the time of searching one set of parameters is about 5 times that of the regression using specified parameter sets. Considering the fact that the time grows as the parameter C becomes larger, actually, the time cost is in fact larger than *n *× 5 × *t*_sin *glepara*_, where *n *represents the number of parameter sets been searched, *t*_sin *glepara *_represent time cost when using one set of specific parameter set. So when the data set is very large, the user has to search in a relatively small range of the parameter sets, to balance the time cost and the performance of SVR impute method.

### Input coding scheme

In the present study, the input vectors of the SVR consist of the (*n *- 1) columns in the profile, and the target output is the prediction of the missing value in the matrix. For example, in the expression matrix D when entry *d*_*i*, *j *_is missing the remaining (*n *- 1) entries in the *i*-th gene expression profile compose the input vector

*v *= [*d*_*i*,1_, *d*_*i*,2_,...,*d*_*i*,*j*-1_, *d*_*i*,*j*+1_,...,*d*_*i*,*n*_]  (11)

All the rows in the expression matrix with non-missing values in the *j*-th position are used to compose the training set, which will be mapped into higher dimensions and construct a model for regression; all the rows with missing values in the *j*-th position were used to compose the testing set. Because the SVR can estimate one missing value in a row at one time, in the case of more than one missing values occurred in one row, the following input coding schemes can be employed, zero-imputing input coding, row-average-imputing input, and orthogonal input.

Zero-imputing input coding scheme imputes the missing values in the input vector with zeros. Row-average-imputing input coding scheme imputes the missing values in the input vectors with the average value of the non-missing values in the row.

Orthogonal input coding scheme is one of the useful input coding schemes those are widely used in machine learning technology such as neural networks and support vector machines. In recent years, it has been successfully used in various fields in biology, such as prediction of protein secondary structure [26], solvent accessibility [27], etc. It is presented as follows. Each value in the input vector is expended to two dimensions. The first dimension is the real value of the input vector, where the missing value is imputed with zeros, and the second dimension is a flag bit, where the missing value is set to be 1 and the others are set to be zeros, thus the length of the input vectors in orthogonal input coding scheme is expanded to 2 × (*n*-1). For example, let (*x*_1_, *x*_2_, *x*_3_, *x*_4_, *x*_5_) denote an expression profile with the length of 5, in which *x*_2 _and *x*_4 _are the missing values. In the calculation of *x*_2_, the zero-imputing input coding scheme, row-average-imputing input coding scheme and orthogonal input coding scheme obtain the input vectors of (*x*_1_, *x*_3_, 0, *x*_5_), (*x*_1_, *x*_3_, *aver*, *x*_5_) and (*x*_1_, 0, *x*_3_, 0, 0, 1, *x*_5_, 0), respectively, where *aver *= (*x*_1 _+ *x*_3 _+ *x*_5_)/3.

## Authors' contributions

XW conceived of the study, wrote program code, analyzed the results and drafted the manuscript. AL participated in programming, helped in analysis and drafting the manuscript. ZHJ helped in analysis and discussion, gave useful comments. HQF guided the study and coordinated the project. All authors read and approved the final manuscript.
